# ROCK ‘n TOR: An Outlook on Keratinocyte Stem Cell Expansion in Regenerative Medicine via Protein Kinase Inhibition

**DOI:** 10.3390/cells11071130

**Published:** 2022-03-27

**Authors:** Giorgia Centonze, Sara Centonze, Luca Ponzone, Enzo Calautti

**Affiliations:** 1 Department of Molecular Biotechnology and Health Sciences, Molecular Biotechnology Center, University of Turin, Via Nizza 52, 10126 Torino, Italy; giorgia.centonze@unito.it (G.C.); luca.ponzone@unito.it (L.P.); 2 Department of Translational Medicine, University of Piemonte Orientale, 28100 Novara, Italy; sara.centonze@uniupo.it; 3 Center for Translational Research on Allergic and Autoimmune Diseases (CAAD), University of Piemonte Orientale, 28100 Novara, Italy

**Keywords:** keratinocyte stem cell, regenerative medicine, cell therapy, mammalian target of rapamycin (mTOR), Rho-associated protein kinase (ROCK), rapamycin, Y-27632

## Abstract

Keratinocyte stem cells play a fundamental role in homeostasis and repair of stratified epithelial tissues. Transplantation of cultured keratinocytes autografts provides a landmark example of successful cellular therapies by restoring durable integrity in stratified epithelia lost to devastating tissue conditions. Despite the overall success of such procedures, failures still occur in case of paucity of cultured stem cells in therapeutic grafts. Strategies aiming at a further amplification of stem cells during keratinocyte ex vivo expansion may thus extend the applicability of these treatments to subjects in which endogenous stem cells pools are depauperated by aging, trauma, or disease. Pharmacological targeting of stem cell signaling pathways is recently emerging as a powerful strategy for improving stem cell maintenance and/or amplification. Recent experimental data indicate that pharmacological inhibition of two prominent keratinocyte signaling pathways governed by apical mTOR and ROCK protein kinases favor stem cell maintenance and/or amplification ex vivo and may improve the effectiveness of stem cell-based therapeutic procedures. In this review, we highlight the pathophysiological roles of mTOR and ROCK in keratinocyte biology and evaluate existing pre-clinical data on the effects of their inhibition in epithelial stem cell expansion for transplantation purposes.

## 1. Introduction

Stratified epithelia provide an essential barrier to the organisms against physical and chemical injuries, dehydration, and pathogens infection. Keratinocyte stem cells play fundamental roles in homeostasis and repair of stratified epithelia throughout the organisms’ lifespan [1], but the nature of the cell populations responsible for the homeostatic regeneration and repair of these tissues in vivo is still under debate. Depending on the specific context, it has been proposed that stratified epithelia regeneration can be driven either by “professional” hardwired stem cells, equipotent progenitor cells, or even differentiated cells resuming stem cell functions upon tissue damage [2,3,4].

Regardless of “semantic” stem cells definitions, engraftment of cohesive sheets of human keratinocytes expanded ex vivo can restore a durable integrity in stratified epithelia compromised by devastating tissue conditions, such as third-degree skin burns or ocular burns that compromise the corneal transparency [5,6,7,8]. Moreover, transgenic cultured epidermal autografts can regenerate a functional epidermal barrier in patients suffering from skin genetic disorders, such as junctional epidermolysis bullosa [5]. Therapeutic grafts obtained by cultured keratinocytes thus contain cells possessing defining stem cell features, such as long-term self-renewal and tissue regeneration ability. Maintenance and/or amplification of these cell populations during the expansion ex vivo of keratinocytes is clearly a prerequisite for the clinical success of such procedures.

Human keratinocytes require proper culture conditions to maintain a full regenerative potential upon transplantation. Optimal results are obtained with a feeder layer (FL) of mitotically inactivated murine J2-3T3 fibroblasts, selected batches of fetal bovine serum, plus a number of additional supplements. Such culture method, first developed by Rheinwald and Green (hereafter R&G) in the mid-1970s, is still regarded as the “gold standard” in clinical settings [9,10]. Notably, R&G conditions do not mimic epidermal homeostasis but rather recapitulate the burst of epidermal regeneration occurring upon wound healing [11]. Under these conditions, the capacity of individual keratinocyte clones to generate secondary clonogenic cultures can be quantified via morpho-functional clonal analysis. Based on this procedure, founder clones can be categorized as holoclones, meroclones, and paraclones [12]. Holoclones possess the greatest proliferative potential and self-renewal ability; paraclones are colonies with short lifespan approaching terminal differentiation, and meroclones have intermediate properties. Upon sub-cultivation, holoclones undergo “clonal conversion”, a process in which they progressively transform into meroclones and eventually paraclones due to the activation of intrinsic differentiation and/or senescence cellular programs. Clonal conversion is promoted by extensive cell manipulation procedures and is further exacerbated by improper culture conditions [13].

Although whether or not meroclones contain stem cells is matter of debate, there is a general consensus that holoclones possess *bona fide* stem cell properties. At the molecular level, holoclones derived from different stratified epithelia, such as the epidermis, the thymic epithelium, and the limbal-corneal epithelium, express elevated levels of the ∆Np63α isoform, a key determinant of keratinocyte cell identity, which is essential for the maintenance of stem cells proliferative potential and survival ability [14,15,16,17]. A very recent study has further unraveled the transcriptional profiles of human epidermal holoclones at the single-cell level [11]. Besides possessing elevated p63 levels, holoclone-forming cells selectively express the FOXM1 transcription factor, a YAP target gene required for long-term self-renewal ability. Moreover, in holoclone-forming cells, gene programs implicated in DNA repair, chromosome segregation, spindle organization, and telomerase activity are overrepresented as compared to meroclones and paraclones.

Of note, keratinocytes with holoclone-like features have been isolated from several other mammalian species and tissues. For instance, in the ocular surface epithelia, holoclone-like colonies have been described in rabbit [18], pig [19,20], rat, calf, and sheep [19]. Moreover, ∆Np63α-bright holoclones have also been described in the murine thymic epithelia [17]. Colonies with holoclone-like features in vitro and tissue-regenerative ability in vivo have also been isolated by rat whiskers [21], dog [22], and murine hair follicle, the latter coinciding with α6^+^CD34^+^ stem cells [23]. Although murine interfollicular keratinocytes do not give rise to the same clonal categories identified in the corresponding human cultures, human holoclone-forming cells and murine epidermal stem cells directly isolated from murine skin express similar subsets of genes [11,24,25], suggesting that holoclones capture a functional status common to stem cells resident of stratified epithelia in several species and contexts.

It is a well-accepted notion that the therapeutic outcomes of keratinocyte autografts depend on the number of holoclone-forming cells in the transplanted epithelial sheets [5,24]. For example, the limbal epithelium, positioned at the boundaries between the conjunctival and the corneal epithelium, is a stem cell niche that provides keratinocyte progenitor cells for the lifelong regeneration of the corneal epithelium; cells isolated from this area exhibit holoclone features in culture [25]. Limbal stem cell deficiency (LSCD) indicates a group of pathological conditions of different origin, such as chemical burns, that compromise the regenerative capacity of the limbal-corneal epithelium and result in corneal opacification and blindness [5]. Transplantation of epithelial sheets generated from limbal keratinocytes expanded ex vivo can restore in the long term the integrity of the corneal epithelium in LSCD patients [5]. A retrospective study on the therapeutic efficacy of limbal autografts indicated that these procedures were clinically successful in about 60–70% of treated patients, and their success was tightly related to the presence of at least 3.0% of holoclone-forming (∆Np63α-bright) keratinocytes in the primary cultures from which the epithelial sheets were generated [9]. Thereafter, the detection of sufficient numbers of ∆Np63α-bright cells in limbal cultures was included as a necessary quality-control step prior to the engraftment of epithelial sheets generated in vitro [26]. Overall, these data indicate that although the success rate of these cellular therapies is relatively high, there is still a margin for their improvement, and a further amplification of holoclones during the expansion of cells ex vivo would likely increase the number of patients that could benefit from these treatments. 

One remarkable example of the power of cell culture manipulation for stem cells fate regulation is provided by pluripotent stem cells. In both mice and humans, cultured pluripotent stem cell lineages can be derived from the inner cell mass cells of pre-implantation embryos (embryonic stem cells, ES cells) [27,28] or generated by the reprogramming of somatic cells through ectopic expression of defined factors (induced pluripotent stem cells; iPS cells) [29]. However, pluripotency and self-renewal are not homogeneously distributed among all cultured cells under standard culture conditions, and a fraction of cells spontaneously differentiate. Notably, dual pharmacological inhibition of the ERK and GSK3 signaling pathways (2i condition) allows the propagation of homogeneous ES/iPS cells populations in a “naïve” pluripotent state equivalent to that of pre-implantation epiblast cells in vivo [29,30,31].

Because culture conditions are also critical for holoclone numbers and functionality [9], these cell populations must respond to environmental cues dictating the choice between self-renewal and exhaustion. Thus, in patients facing a decline in the endogenous keratinocyte stem cell pools as a consequence of aging, trauma, or diseases, the ex vivo inhibition of differentiation or stress-response pathways that drive stem cells exhaustion may restore holoclone numbers above the threshold required for a successful outcome of cultured keratinocyte autografts. 

In this manuscript, we review preclinical evidence indicating that the pharmacological inhibition of the mammalian target of rapamycin (mTOR) or the Rho kinase (ROCK) pathways, by favoring different aspects of keratinocyte stem cell biology, might be applied either alone or in combination to improve the efficacy of cell-based therapeutic transplants in regenerative medicine.

## 2. mTOR Signaling

**Figure 1:**
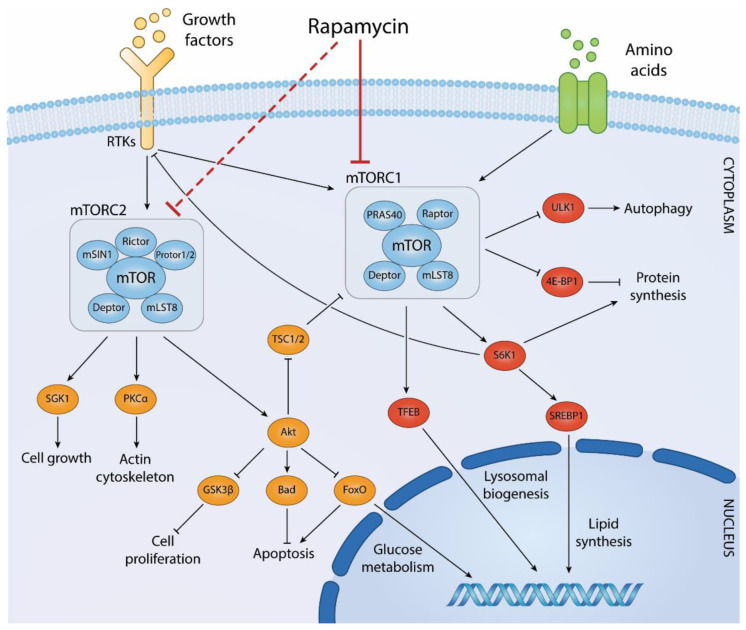
Biological processes regulated by mTOR complexes and downstream effector pathways. Both mTORC1 and mTORC2 are activated downstream of growth factors binding to cell surface receptors, such as receptor tyrosine kinases (RTKs), while mTORC1 receives stimulatory inputs also by amino acids. The diagram reports mTOR binding partners within mTORC1 and mTORC2 as well as their best-established downstream effector molecules in the indicated biological processes. Within the nucleus, signaling molecules downstream of mTOR signaling regulate gene expression programs involved in glucose metabolism, lysosomal biogenesis, and lipid synthesis. The diagram includes the negative feedback emanating from the mTORC1 effector S6K1 on RTKs downstream signaling. The allosteric mTOR inhibitor rapamycin primarily inhibits mTORC1 but also mTORC2 upon prolonged exposure of cells to the drug.

mTOR belongs to the Phosphatidylinositol 3-kinase-related kinases (PIKK) family of serine/threonine protein kinases and regulates a plethora of biological processes, such as cell growth, proliferation, protein and lipid synthesis, cancer, aging, lysosome biogenesis, and stress response. mTOR nucleates at least two distinct multi-protein complexes, namely mTOR complex-1 (mTORC1) and mTOR complex-2 (mTORC2) [32]. Figure 1 summarizes the regulation of mTOR complexes and their downstream signaling events involved in specific cellular functions.

### 2.1. mTORC1

mTORC1 is composed of five protein subunits (including mTOR): two subunits shared with mTORC2, namely mLST8 (mammalian lethal with Sec13 protein 8) [33] and DEPTOR (DEP-domain-containing mTOR-interacting protein) [34], and two exclusive subunits, Raptor (regulatory-associated protein of mTOR) [35] and PRAS40 (proline-rich Akt substrate 40kDa) [36]. 

When activated by upstream growth factor signaling and nutrients (amino acids), mTORC1 promotes protein synthesis through phosphorylation of two key protein substrates: 4E-BP1, which then dissociates from eIF4E, leading to initiation of protein translation, and S6K1 protein kinase. Upstream signaling by amino acids largely relies on activation of Rag-dependent mechanisms, which lead to localization of mTORC1 at the lysosomal surface, where it becomes activated by GTP-bound Rheb [37]. The amount of GTP-bound, active Rheb is negatively regulated by the GAP activity of tuberous sclerosis -1 and -2 (TSC1/TSC2) protein complexes that stimulate the intrinsic GTPase activity of the protein. Growth factors signaling, via activation of Phosphoinositide 3-kinase (PI3K) pathway, leads to phosphorylation of TSC2 by Akt (or other kinases) at regulatory residues, releasing the inhibitory activity of TSC1/TSC1 on Rheb. This leads to the accumulation of the GTP-bound form of the protein with subsequent activation of mTORC1 at the lysosomal surface. Since both growth factors and nutrients converge on the activation of mTORC1, starvation acts as a potent physiological inhibitor of this signaling complex.

mTORC1 regulates de novo lipid biosynthesis and lysosomal biogenesis [38]. Importantly, mTORC1 suppresses ULK1 (Unc-51-like kinase)-dependent autophagy, and therefore, physiological mTORC1 inhibition potently activates autophagy [39,40].

On the other hand, sustained mTORC1 activation induces negative feedback mechanisms that desensitize cells to growth-factors-dependent stimuli. The best known example is the mTORC1-dependent degradation of the IRS1 adaptor protein, a protein that couples PI3K activation to insulin/IGF (IIS) signaling. Following IIS stimulation, IRS1 is targeted to proteasomal degradation by phosphorylation by S6K1 [32], with a subsequent attenuation of IIS signaling. Moreover, other mTORC1-dependent mechanisms of growth factor signaling inhibition have been described [32].

Overall, these negative feedback mechanisms limit in time the cellular responses to growth factors, and their abrogation via mTORC1 inhibition favors activation of the PI3K to Akt signaling, resulting in the stimulation of cell survival [41].

mTORC1 is also inhibited by different forms of cellular stress. Prolonged mTORC1 signaling activation leads to the erosion of cellular energy stores due to consumption of ATP during anabolic processes. Therefore, intrinsic to the response of cells to stress is the limitation of energy expenditures to favor survival versus growth via attenuation of mTORC1 signaling activity [42].

For instance, genotoxic stress leads to a p53-dependent mTORC1 inhibition [43]. p53 limits mTORC1 signaling via its direct target genes, Sestrin1- and -2, that inhibit mTORC1 lysosomal localization and activation [44,45]. Additionally, mTORC1 is also inhibited under hypoxic conditions [46], at least in part via HIF1α-dependent induction of the mTORC1 inhibitory protein Redd1 [47].

The Forkhead box O (FOXO) family of transcription factors plays positive roles in lifespan extension, resistance to oxidative stress, and stem cell maintenance [48]. By driving the expression of antioxidant target genes, such as MnSOD and Catalase, FOXOs promote tolerance to oxidative insults in several animal models. FOXO transcriptional activity is directly stimulated by oxidative stress [49], while it is antagonized by IIS signaling [50]. As a part of their stress-adaptive transcriptional program, FOXOs induce expression of mTORC1 inhibitory genes, such as TSC1 [51] and Sestrin3 [52], and this function is conserved from yeast to mammals [42]. Interestingly, both FOXO activation and mTORC1 inhibition increase animal lifespan in several model organisms [53,54]. Therefore, it seems likely that the pro-longevity roles of FOXOs rely, at least in part, on attenuation of mTORC1 signaling. 

### 2.2. mTORC2

mTORC2 complex is composed of mTOR, mLST8, DEPTOR, Sin1/MAPKAP1 (mammalian stress-activated MAPK-interacting protein 1), Rictor (rapamycin-insensitive companion of mTOR), and Protor1/2 (protein observed with Rictor 1/2) [32]. The best established function of this complex is the phosphorylation of AGC kinases, such as Akt/PKB, SGK1 (serum/glucocorticoid-regulated kinase 1), and PKCα (and possibly other PKC isoforms). mTORC2 phosphorylates Akt at Serine 473, located in the hydrophobic regulatory motif, which leads to full Akt kinase activation. Akt regulates cell survival, proliferation, and energetic metabolism via multiple protein substrates, such as TSC2, GSK3β (glycogen synthase kinase 3β), and FoxO proteins. SGK1 regulates ion transport and apoptosis [55], while PKCα controls cytoskeleton organization and cell motility [56]. Like mTORC1, also mTORC2 is involved in the regulation of cell metabolism in several tissues by promoting glycolysis, lipid synthesis, and amino acid transport [32]. 

mTORC2 regulation is primarily achieved by growth factor signaling through the PI3K pathway. The link between growth factor signaling, PI3K, and mTORC2 is provided by its integral component, Sin1, which possesses a pleckstrin homology domain that inhibits mTORC2 in the absence of active PI3K signaling. Binding to PI3K lipid products phosphatidyl inositol (3,4,5) triphosphate (PIP3) at the plasma membrane releases this inhibition and fosters interaction between mTORC2 and its Akt protein substrate, which is also recruited at PIP3-enriched membrane domains. Cross-phosphorylation between Akt and mTORC2 further modulates their activation and subcellular distribution [57,58].

Interestingly, distinct subcellular pools of mTORC2 at the plasma membrane, mitochondria, and endosomal vesicles have been described, which possess a differential dependence on growth factor signaling and PI3K activity [57]. 

### 2.3. Roles of the mTOR Signaling in Development, Stem Cell Regulation, and Aging

The biological functions of mTOR can be investigated either via direct pharmacological inhibition or by genetic ablation of genes encoding mTOR or its key molecular partners. 

The most extensively used mTOR inhibitor is rapamycin, a macrolide that causes dissociation between mTOR and RAPTOR proteins within the mTORC1 [32] and which has long been considered as an mTORC1-selective inhibitor. It is now well established, however, that prolonged rapamycin treatment also inhibits mTORC2 due to the progressive sequestration of newly-formed mTOR moieties [41]. Rapamycin and its “rapalogs” derivatives, however, only partially block the activity of mTORC1, having a limited effect on the phosphorylation of selected targets, such as 4EBP1, the phosphorylation of which is essential for initiating CAP-dependent mRNA translation at the ribosome [59]. A second class of mTOR inhibitors, including Torin1 and PP242, also known as Torkinib, has been developed. These compounds function as ATP-competitive inhibitors of the mTOR protein kinase, blocking both mTORC1 and mTORC2 downstream signaling, thus displaying a broader range of action than rapamycin [60,61]. Third-generation mTOR inhibitors have also been developed. These latter compounds (such as Rapalink 1) combine the high affinity of rapamycin for mTORC1 with the spectrum of action of mTOR kinase inhibitors [62]. Another pharmacological compound reported to inhibit mTORC1 from upstream is the recently developed small molecule (NR1), which represents a selective Rheb inhibitor [63]. Therefore, when interpreting data based on mTOR pharmacological inhibition, it is essential to take into account both the mechanisms of action and the downstream targets of each specific class of drugs.

Complete disruption of the mTOR gene in mice leads to early developmental arrest at the pre-implantation blastocyst stage, with inner cell mass abnormalities and failure to derive ES cell lines from *mTOR*^−/−^ blastocysts [64,65]. Homozygous deletion of Raptor and Rictor in mice leads also to severe developmental defects, with Raptor deficiency causing early embryonic lethality, closely resembling mTOR deficiency, while Rictor ablation is embryonically lethal at a later developmental stage (E 10.5) due to severe defects in vascular development [66]. These studies indicate essential roles of mTOR in growth and proliferation of early embryos and ES cells [65].

In somatic stem cells, mTORC1 signaling activity varies depending on their functional state and degree of commitment towards differentiation. In many types of stem cells, low levels of mTORC1 activity are needed to maintain self-renewal capability, while an increase in mTORC1 signaling is generally observed upon commitment of cells to differentiation. It seems likely that mTORC1 activation in stem cells marks the change of state from quiescence to activation by favoring anabolic processes required during progenitor cell expansion and subsequent differentiation [67,68,69,70,71]. This concept is supported by studies carried out on neural stem cells [72,73], mammary stem cells [74], hematopoietic stem cells (HSCs) [75,76,77], and germline stem cells [78,79]. Collectively, sustained attenuation of the mTORC1 signaling pathway prevents stem cell loss and/or favor their long-term maintenance in several tissues and developmental contexts both in vitro and in vivo. 

While it is well established that mTORC1 plays important roles in the regulation and maintenance of various stem cell types, the roles of mTORC2 in this context are still poorly investigated. However, it has been reported that mTORC2 regulates differentiation of murine ES cells to mesoderm independently of its role in the activation of mTORC1 signaling from upstream [80]. Moreover, in murine mesenchymal stem cells (MSCs), mTORC2 promotes osteoblast differentiation and suppresses adipocytes differentiation, while mTORC1 plays an opposite role [81].

Over the past several years, there has been accumulating evidence demonstrating that mTOR inhibition via rapamycin (or rapalogs), extends lifespan in yeast, worms, flies, and mice, indicating that attenuation of mTOR signaling promotes longevity in a wide range of species [82].

The mechanisms underlying rapamycin pro-longevity effects are not completely understood, but mTOR signaling plays key roles in negative regulation of autophagy, which in turn is linked to lifespan extension [83]. Autophagy is a process that allows cells to adapt to metabolic stress through degradation and recycling of intracellular components to generate macromolecular precursors and produce energy [84]. Of note, autophagy plays critical roles in stem cell quiescence, activation, differentiation, and self-renewal. Moreover, defective autophagy in stem cells contributes to aging, degenerative diseases, and generation of cancer stem cells [84]. It is believed that preservation of stem cell functionality is likely one major factor behind the pro-longevity effects of rapamycin [71].

mTORC1 suppresses autophagy primarily by inhibiting ULK1, the autophagy-initiating kinase, through direct phosphorylation at Serine 757, and mTORC1 inhibition acts as a sufficient trigger of autophagy in many cellular contexts [39]. 

Telomere length and telomerase activity are key determinants of stem cell maintenance and aging. It has been recently shown that mTOR inhibition via rapamycin does not promote telomere maintenance in normal mice, and telomerase-deficient mice show hyperactivation of mTOR [85]. Interestingly, prolonged rapamycin treatment does not exert a life-extending activity in mice with a dramatic telomere shortening but rather anticipates the death of the animals, suggesting that mTOR activation provides a survival advantage to cells and tissues with shortened telomeres, while its inhibition becomes lethal in this context.

### 2.4. mTOR Signaling in Keratinocyte Stem Cells Fate Determination

Stratified epithelia homeostasis is maintained by a finely tuned balance between keratinocyte proliferation and differentiation. The epithelial portion of the skin is composed by the interfollicular epidermis (IFE), hair follicles (HFs), and sebaceous glands (SGs) subcompartments, in which “private” pools of stem/progenitor cells maintain tissue homeostasis in their respective domains but also contribute to other districts’ regeneration of upon injury [86].

In the attempt to define the roles of Wnt signaling in skin carcinogenesis, Castilho and colleagues generated a transgenic mouse that allowed conditional overexpression of Wnt1 in the epidermal and HF compartments [87]. Consistent with the roles of Wnt signaling in HF morphogenesis and cycling, the animals displayed a dramatic hair follicle growth early in life upon the induction of the transgene. However, the initial proliferative burst of HFs was followed by severe alopecia development later in life, caused by premature senescence of CD34^+^ bulge HF stem cell populations (HFSCs). [87]. Such cell population are quiescent throughout the resting phase of the HF cycle (telogen) but become rapidly activated by Wnt proteins to initiate the growing phase of the HF cycle (anagen). The authors realized that sustained Wnt1 expression also caused a prolonged activation of mTORC1 and that a pharmacological inhibition of mTOR with rapamycin parallel to Wnt1 overexpression restored quiescence and prevented HFSCs loss. The underlying mechanism of stem cells regulation by Wnt and mTORC1 relies on the capacity of GSK3 kinase, an endogenous suppressor of Wnt signaling, of enhancing the GTPase activity of TSC1/TSC2 complexes on Rheb, which ultimately results in mTORC1 inhibition and promotion of HFSC quiescence. Conversely, by inhibiting GSK3, Wnt1 leads to mTORC1 activation [88]. The HFSC senescent phenotype resulting from hyperactivation of mTOR has been interpreted as a failsafe mechanism limiting the effects of sustained pro-oncogenic stimulus (such as Wnts), similar to what has been observed in HSCs subjected to conditional deletion of the PTEN tumor-suppressor gene. PTEN is the main negative regulator of the PI3K/Akt/mTORC1 axis, and its deletion causes a transient expansion followed by depletion of HSCs, which can be prevented by the concomitant treatment of mice with rapamycin [89]. 

It has been later demonstrated that, in the context of cultured human oral keratinocytes, rapamycin treatment can also prevent the onset of cell senescence, a major cause of SC depletion induced by either extensive cell propagation or exposure of cultures to ionizing radiations [90]. Mechanistically, the protective effect of rapamycin was found to rely on increased protein levels of mitochondrial superoxide dismutase (MnSOD), which raises the tolerance of cells to the elevated ROS levels that are induced by these stressors. Remarkably, the authors also found that a pre-treatment of mice with rapamycin preserved the integrity of the oral mucosa in vivo to clinically relevant doses of gamma radiation. However, a recent work based on ID-seq technology coupled to a screening of a library of known kinase inhibitors [91] identified mTOR inhibition as an important event required for terminal differentiation of human epidermal keratinocytes, induced by EGFR inhibition under serum- and feeder-free conditions. This suggests that mTOR signaling plays a multi-faceted role in the keratinocyte differentiation program.

In cultured human limbal keratinocytes (LK), Saoncella et al. found that the Akt/FoxO axis plays a key role in the regulation of LK stem cells (LKSCs) self-renewal by determining the mTORC1 signaling outputs [92]. In this cellular context, Akt1 and Akt2 isoforms have opposite roles in LKSCs maintenance, as attenuation of Akt2 signaling strongly enhances LKSCs maintenance ex vivo, whereas Akt1 depletion anticipates their exhaustion. These isoform-specific roles rely on the distinct subcellular distribution of the two Akt isoforms. Akt2 is the main nuclear Akt isoform that phosphorylates FoxO1 transcription factor in this cellular district, leading to its nuclear export and inhibition. FoxO1 inhibition, in turn, leads to loss of LK self-renewal, which largely depends on an excessive activation of mTORC1 signaling. Upon Akt2 downregulation, FoxO1 becomes stabilized within the nucleus and reduces mTORC1 signaling via transcriptional activation of the TSC1 gene, encoding for one essential component of the TSC1/TSC2 mTORC1 inhibitory complex. mTORC1 signaling attenuation via TSC1 resulted crucial for LKs self-renewal since TSC1 knockdown abolished the increase in self-renewal and clonogenic capacity induced by Akt2 silencing with subsequent stem cell depletion. In agreement to these findings, another study indicated that in primary human LKs, rapamycin treatment promotes a higher long-term proliferative potential and increased expression of LKSCs markers along with a decrease in basal cell-proliferation rates [93]. Collectively, these results indicate that genetic or pharmacologic attenuation of mTORC1 signaling counteracts the onset of senescence in cultured limbal-corneal epithelial cells and suggest that rapamycin or its analogues could be potentially applied to foster LKSCs maintenance/amplification during the therapeutic expansion of LKs. 

### 2.5. Effects of Genetic Disruption of mTOR Signaling Components in Epidermal Development

While a partial mTOR blockade, such as that induced by rapamycin, preserves stratified epithelia stem cells number and functionality in various in vitro and in vivo contexts, a drastic suppression of mTOR (and in particular of mTORC1 signaling) during epidermal development severely compromises tissue morphogenesis and the formation of a functional epithelial barrier. 

In fact, Ding and colleagues reported that the tissue-specific deletion of mTOR gene in the developing murine epidermis results in severe defects in epidermal morphogenesis and stratification subsequent to abnormalities in the epidermal differentiation program [94]. Epidermal-specific mTOR-KO mice die shortly after birth for dehydration caused by a defective epidermal barrier. This phenotype was also recapitulated by the epidermal-specific deletion of the essential mTORC1 component Raptor. Similarly, Asrani and colleagues [95] showed that defective mTORC1 signaling, achieved by epidermal-specific deletion of either Raptor or Rheb, compromises epidermal morphogenesis and skin barrier function, with dramatic defects in desmosomal cadherin function that are necessary for epidermal stratification and integrity. Interestingly, the cell-adhesive abnormalities induced by Raptor ablation or by pharmacological inhibition of mTORC1 signaling were found associated with enhanced TGF-beta-dependent activation of ROCK1 protein kinase. Pharmacological inhibition of ROCK was sufficient to restore proper keratinocyte cell–cell adhesive functions of Raptor-KO keratinocytes in vitro. 

The roles of mTORC2 in epidermal development were investigated via epidermal-specific deletion of Rictor. Ding and coworkers reported that Rictor/mTORC2 deficiency causes moderate epidermal thinning with mild cell differentiation defects during embryonic and early post-natal life, without obvious skin or hair follicle phenotypes in young adult animals [94]. Interestingly, in the developing embryo, the authors found that mTORC2 deficiency causes abnormal mitotic polarization of basal cells, with an increase of symmetric divisions at the expenses of asymmetric cell divisions, the latter necessary for the initiation of epidermal stratification during development. This phenomenon can account in part for the reduced stratification of neonatal Rictor-deficient epidermis and likely depends on abnormalities in actin cytoskeleton dynamics. The authors proposed that the differentiation abnormalities of Rictor-KO epidermis depend on attenuation of Akt signaling, which plays important roles in keratinocyte differentiation [96], whereas defective PKC signaling accounts for cytoskeletal defects [94]. However, these possibilities were not further investigated.

Tassone and colleagues also generated an epidermal-specific Rictor-KO mouse. They found that in addition to a hypoplastic epidermis and mild differentiation defects in newborn animals, Rictor deficiency leads to profound changes in keratinocyte metabolism. In fact, Rictor-KO keratinocytes display decreased expression of genes involved in lipid metabolism and display a rewiring of their energetic metabolism from glycolysis to glutaminolysis. Importantly, these metabolic changes were associated with an increased resistance to several cellular stressors that converge on the production of cellular ROS, which in turn activate stress protective cellular programs (mitohormesis). In fact, Rictor-deficient keratinocytes have increased levels of mitochondrial ROS, which depend on their increased glutamine consumption in the glutaminolysis pathway. Notably, cultured Rictor-deficient cells are also protected from senescence and prone to spontaneous immortalization [97].

Rictor/mTORC2 functions were also found upregulated in the epidermis of aged mice, suggesting a role in skin aging [98]. Although none of these studies has addressed the specific role of Rictor in IFE stem cells, these data collectively indicate that Rictor deficiency/mTORC2 inhibition may favor two key aspects crucial for IFE keratinocyte SCs: protection from senescence and the capacity to switch between asymmetric and symmetric cell division. This is particularly relevant considering that all known mTOR inhibitors available to date can block the activity of both mTOR complexes upon prolonged exposure [41].

However, Rictor/mTORC2 deficiency was recently found to impair hair follicle regeneration in aged mice [99]. Mechanistically, this was due to the increased glutamine metabolism of follicular keratinocytes that interferes with the return of keratinocyte progenitor cells into the hypoxic HFSC niche at the end of the anagen phase. This prevents these cells from resuming a stem cell state and replenishing the HFSC pools in the long term.

## 3. ROCK Pathway

**Figure 2:**
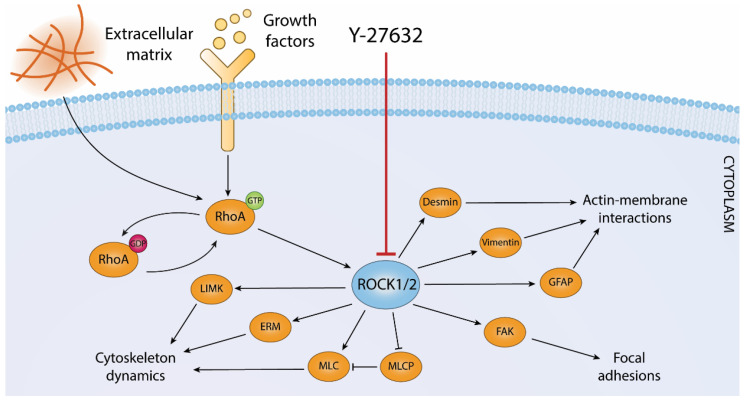
ROCK signaling in the regulation of cytoskeleton structure and dynamics.

Rho-associated protein kinases (ROCKs) belong to a serine-threonine kinase family mainly involved in the regulation of cell adhesion, motility, and cytoskeleton dynamics. ROCKs are well-known downstream effectors of Rho GTPases, and their functions include actin filaments organization, contractility regulation, stress fibers association, focal adhesions formation, cytokinesis, migration, and apoptosis [100,101]. The Rho-associated kinase family is composed by two isozymes, named ROCK1 and ROCK2, which share a common ATP binding pocket and partially distinct protein functions and distribution; for instance, ROCK1 is mostly expressed in liver, lung, spleen, kidney, and testis, while ROCK2 is found primarily in brain and skeletal muscle [102]. Both ROCK1 and ROCK2 phosphorylate various downstream protein substrates, such as LIM kinase, myosin light chain (MLC), and MLC phosphatase (MLCP), which are involved in cytoskeleton dynamics, highlighting the importance of ROCKs as key regulators of cell adhesion, migration, and contractility. In addition, ROCK phosphorylates focal adhesion kinase (FAK) and promotes its activation by Pyk2 [103], thereby mediating assembly and turnover of focal adhesions [104]. Moreover, ROCKs phosphorylate ezrin-radixin-moesin (ERM) family of proteins and the Na/H^+^ antiporter NHE-1, thereby regulating actin-membrane interactions [105,106]. Finally, ROCKs regulate the formation of intermediate filament structures by phosphorylating desmin, vimentin, and glial fibrillary acidic protein (GFAP) [107]. Figure 2 provides a schematic representation of ROCK signaling network in the regulation of cytoskeleton dynamics. 

ROCK kinases respond to upstream stimulatory inputs originated by growth factors and contact with the extracellular matrix components through GTP-bound, active RhoA (RhoA-GTP). A subset of ROCK 1/2 effector molecules that play key roles in the regulation of actin cytoskeleton dynamics, cell shape, and mechano-transduction is indicated (see text for details). Y-27632 is a potent and specific ATP-competitive inhibitor of ROCK 1/2.

### 3.1. Roles of ROCK Signaling in Development and Stem Cell Regulation

Murine models deficient of either ROCK isoform do not show obvious epidermal defects or phenotypes. In fact, about 90% *Rock2* knockout mice die in utero due to extensive thrombus formation, and few survivors are born as runts and develop without obvious abnormalities and are fertile [108].

*Rock1* knockout mice instead are born alive, but the majority of mice die immediately after birth due to omphalocele and exhibit eyelids open at birth. The majority of surviving *Rock1*^−/−^ mice subsequently develop normally, and with the exception of the eye lesions, the adult mutant animals are fertile and apparently healthy. Moreover, skin wound healing was also found normal in ROCK1-deficient mice, and primary keratinocytes isolated from these animals exhibited mild defects in actin reorganization in response to EGF [109].

*Rock1*^−/−^*Rock2*^−/−^ embryos die in utero between E3.5 and E9.5, and embryonic lethality around E9.5 is observed also in *Rock1*^+/−^/*Rock*2^−/−^ mice or *Rock1*^−/−^/*Rock*2^+/−^ mice due to impaired vasculature development in the yolk sac (for review, see [104]). 

These data suggest that the deficiency of individual ROCK isoforms do not impact dramatically epidermal development and homeostasis. It is therefore possible that ROCK1 and -2 isoform play redundant roles in murine skin development and homeostasis. However, the generation of epidermal-specific *Rock1*^−/−^*Rock2*^−/−^ double-knockout mice has not yet been described.

Stem cells are highly sensitive to mechanical forces that regulate their biology [110]. By participating to a mechano-transduction signaling network, ROCKs play a role in stem cell regulation and differentiation. For instance, McBeath and colleagues demonstrated that in human MSCs, RhoA-ROCK signaling receives inputs both by cell shape and soluble factors and regulates stem cell lineage commitment via changes in cytoskeletal tension [111]. Consistently, Saidova et al. reported that MSCs proliferation and differentiation are regulated by RhoA/ROCK signaling via modulation of cell shape and mechano-transduction [112], and it has also been reported that RhoA-ROCK activation via non canonical Wnt pathway enhances human mesenchymal stem cells lineage commitment via mechanical forces [113]. Moreover, ROCK functions in cell adhesion and actin dynamics contribute to cell fate determination in human adipose stem cells [114]. Additionally, rabbit limbal epithelial stem cells proliferation has been reported to be regulated by activation of Wnt11/Fzd7/ROCK pathway upon adhesion to fibronectin [115].

Several lines of evidence suggest that ROCK signaling inhibition enhances stem/progenitor cell functions in vitro and can inhibit the occurrence of cell senescence. 

The first evidence of a beneficial role of ROCK inhibition for stem cell maintenance comes from the observation of a protective role towards apoptosis/anoikis in human ES cells. Human ES cells are highly sensitive to detachment from the substrate, and this greatly limits their survival under clonogenic conditions. Watanabe and colleagues demonstrated that treatment with the Y-27632 ROCK inhibitor reduces apoptosis and increases the cloning efficiency in ES cells [116]. Similarly, addition of Y-27632 allows the survival of isolated Lgr5-positive intestinal crypt stem cells and the subsequent establishment of intestinal organoid cultures [117]. Thereafter, ROCK pharmacological inhibition with Y-27632 has been used extensively for enhancing the survival ex vivo of several stem cell types, including neuronal precursor [118], and for establishing salivary organoid cultures [119]. Additionally, ROCK inhibition improves the survival and cultivation of human iPS cell-derived cardiomyocytes after dissociation [120], suggesting that the protective effect of ROCK inhibitors is not restricted to stem/progenitor cells. From an applicative standpoint, ROCK inhibition is currently used for protecting different stem cell types from suspension-induced death during their manipulations.

Whereas the pro-survival role of ROCK inhibition seems to hold true across multiple stem cell types, the consequences of ROCK pathway inhibition in stem/progenitor cell fate determination are multifaceted and highly context dependent.

Concerning pluripotent stem cells, one study in human iPS cells showed that mechanical stretching of cells activates RhoA, alters the alignment of actin fibers, and lowers the expression of pluripotency genes, such as Nanog, POU5f1, and Sox2. In this context, treatment with Y-27632 enhanced Akt phosphorylation and restored a normal expression of pluripotency markers, thereby counteracting the effects of mechanical strain [121]. These findings suggest a role for Rho/ROCK pathway as a negative regulator of pluripotency and Akt signaling in response to mechanical cues although the mechanistic link between ROCK and Akt activation was not investigated. However, another study on human iPS cells reported that prolonged treatment with Y-27632 primes the commitment of cells towards the mesodermal lineage while antagonizing ectodermal fate specification [122]. ROCK inhibition was also shown to promote differentiation of human ES cells into neural crest-like progenitors [123]. Differences in pluripotent cell culture conditions, inhibitor dosage, and timing of inhibition can possibly account for these apparent discrepancies. In murine ES-cells-derived Flk1^+^ mesodermal precursor cells, ROCK inhibition promotes differentiation and expansion of endothelial cells [124]. In multipotent adipose-derived stromal/stem cells, ROCK inhibition favors the adipogenic fate at the expenses of the osteogenic one [114]. 

### 3.2. Effects of ROCK Inhibition in Keratinocytes

It has been reported that both RhoA and its downstream effector ROCK are both activated during the execution of the keratinocyte terminal differentiation program [125,126]. The effects of RhoA/ROCK activation on keratinocyte differentiation are rather complex since RhoA is activated early on during murine keratinocyte differentiation, and this event is necessary for the establishment of cadherin-dependent adhesion and keratinocyte stratification [125]. However, expression of active RhoA in keratinocytes has been reported to have both inhibitory and stimulatory effects on differentiation depending on the specific experimental context (e.g., murine vs. human, extracellular calcium concentration, differentiation-inducing stimuli) and the differentiation parameters that have been analyzed [126,127]. Moreover, adding to this complexity, RhoE, a constitutively active Rho protein that inhibits RhoA/ROCK signaling, was also found induced during keratinocyte differentiation [128]. Intriguingly, RhoE expression appears to be positively regulated by ROCK1 [129].

Overall, endogenous RhoA activation is likely required for triggering some aspects of keratinocyte differentiation in a subcellular compartmentalized fashion, while both a sustained RhoA activation or inhibition may cause a delay or an acceleration of the keratinocyte differentiation program depending on the specific context. Of note, ablation of RhoA in the murine epidermis is compatible with normal epidermal morphogenesis and maintenance, suggesting that other Rho isoforms (like RhoB and -C) likely compensate for the loss of RhoA.

Regarding isoform-specific functions of ROCKs, by using the HaCaT immortalized keratinocyte cell line, it has been proposed that ROCK1 and -2 play opposite roles, with the former as an inhibitor and the latter an agonist of keratinocyte differentiation [130]. However, this rigid dichotomy is likely an oversimplification, as other studies on primary keratinocytes suggest that both ROCK1 and -2 can promote different aspects of the differentiation program [131].

Several lines of evidence indicate that pharmacological inhibition of ROCKs can extend the lifespan of cultured human keratinocytes. Chapman and colleagues first described that treatment with Y-27632 promotes primary keratinocytes proliferation and causes a conditional immortalization in cells cultured in R&G conditions. Importantly, keratinocytes subjected to sustained ROCK inhibition retain the potential to differentiate and give origin to a normal stratified epithelium upon drug withdrawal [132]. Keratinocyte lifespan extension was not coupled with telomeres elongation, and telomeres shortening was not abolished upon serial sub-cultivation of cells but rather stabilized when telomeres reach a critical threshold. Moreover, keratinocytes maintained a normal karyotype despite extensive passaging in culture. In a following study, the same group performed a more detailed analysis of the impact of ROCK inhibition on keratinocytes lifespan. They showed that both neonatal and adult keratinocyte lifespan extension can be achieved via different ROCK pharmacological inhibitors, and this effect can be observed also in epidermal cells isolated from other mammals [133]. Global gene expression analysis of Y-27632-treated cells indicated that the main effect of ROCK inhibition is the interruption of the keratinocyte differentiation program as highlighted by the reduced expression of genes involved in epidermal keratinization and differentiation coupled with upregulation of genes involved in cell division and proliferation. Importantly, despite slightly higher levels of ΔNp63α and a modest decrease in expression of genes involved in NOTCH signaling, Y-27632-treated cells did not show significant changes in the levels of genes associated with keratinocyte stemness. The authors describe the effects of ROCK inhibition as almost immediate and reversible: even cells approaching the end of their replicative cycle return to proliferate when Y-27632 is added to the culture medium, but they eventually reach senescence upon its removal [131,133]. 

The effects of ROCK inhibition on the maintenance of the cell-proliferative capacity were confirmed also in another study on human neonatal epidermal keratinocytes. These cells showed an increased lifespan and augmented proliferative capacity when maintained in low-calcium culture conditions in the presence of Y-27632; when the inhibitor is removed, and calcium concentration is increased, keratinocytes differentiate even after prolonged cultivation in Y-27632/low-calcium conditions. [134]. Moreover, in a recent work, Zhang and colleagues showed that low-calcium condition coupled with PAK1-ROCK-Myosin II and TGF-β signaling inhibition enhances the expansion of stem and progenitor cells from different epithelial tissues [135]. ROCK pharmacological inhibition via Y-27632 has also recently reported to improve the expansion of mammalian airway epithelial cells [136].

Different mechanisms have been proposed to underlie the inhibition of keratinocyte differentiation triggered by ROCK pharmacological blockade. However, a consensus on the mechanistic basis of this effect is currently missing. As NOTCH signaling activation plays a key role in keratinocyte differentiation [137], Yugawa and colleagues have shown that ROCK activation can occur downstream of noncanonical NOTCH signaling in keratinocytes and that ROCK inhibition suppresses differentiation triggered by ectopic expression of activated NOTCH1 [131]. Although it has been shown that ROCK activation occurs independently of NOTCH transcriptional activity, the molecular basis of this process is presently unclear. Substantial evidence suggests that the effect ROCK signaling inhibition on keratinocyte differentiation may depend on its functions in the regulation of actin cytoskeleton dynamics. Cytoskeletal reorganization can be induced by different extracellular signals, such as chemokines, growth factors, and characteristics of the substrate, which lead to F-actin structure rearrangements and actin dynamics modulation (see for review [138]). Cell shape as well as the balance between monomeric and filamentous actin can dictate the choice between proliferation and differentiation by impinging on keratinocyte transcriptional programs. In particular, G-actin monomers bind to the megakaryocytic acute leukemia protein and inhibit serum response factor (SRF) transcriptional activity, with a subsequent reduced expression of its direct target genes c-fos and Junb that are required for epidermal differentiation [139].

In the context of keratinocyte clonal growth in R&G conditions, it has been shown that clonal conversion parallels with the reorganization of actin filaments. In growing colonies, actin is radially distributed and EGF exposure leads to an increase in colony size, while terminal colonies present a circumferential distribution of actin and shrink upon EGF treatment.

The use of ROCK inhibitor Y-27632 levels the response of both types of colonies to EGF [140]. However, in agreement with data by Chapman and colleagues, although Y-27632 treatment induces a radial organization of actin in terminal colonies similar to that of the expanding ones, this does not prevent their differentiation commitment. 

By using high-definition time-lapse microscopy system to image clonal cultures of human neonatal foreskin epidermal keratinocytes, the group of Philip H. Jones reconstructed the proliferative history at the single-cell level within hundreds of keratinocyte colonies [141]. The authors proposed that in primary keratinocytes holoclones, meroclones and paraclones reflect the stochastic fluctuation of cells between two proliferating modes, namely expanding and balanced, rather than a “hardwired” hierarchy. In the expanding mode, cell divisions give rise to two proliferating cells, whereas in the balanced mode, one proliferating and one non-proliferating cells are generated.

Holoclones would therefore represent colonies in which during the first days of culture, the proliferative divisions predominate; paraclones instead represent colonies in balanced mode of division and meroclones a mixture of cells in balanced and expanding mode.

The authors found that cells switch from an expanding to a balanced proliferation mode in the center of large expanding colonies, where confluency is attained. However, when the center of the colony is wounded with a scratch, cells that were initially in balanced mode switch back to an expanding one. Importantly, ROCK inhibition with Y-27632 is sufficient to restore an expanding mode in the center of the colony despite confluency. Regardless of whether holoclones represent the product of stochastic or predetermined division modes, this study confirms that ROCK pharmacological inhibition represents a powerful strategy to maximize the proliferative capacity of keratinocyte clonal cultures by forcing cells in a symmetric, proliferative division mode.

### 3.3. Conditional Reprogramming (CR)

The combinatorial use of FL (typically, irradiated 3T3-J2 mouse fibroblasts) and the ROCK inhibitor Y-27632 has been thereafter referred to as Conditional Reprogramming. Overall, compared to other methods for culturing primary epithelial cells, this protocol is rapid and efficient to obtain an unlimited propagation of human cells of both normal and neoplastic origin (for review, see [142,143]. 

CR presents several advantages over other methods of cell immortalization, such as the constitutive expression of viral proteins, e.g., SV40 large tumor antigen, such as the absence of genetic alterations and the reversible effects on long-term cell expansion. CR thus requires the synergy of multiple factors, including molecules released by feeder cells, which, by preventing cell death and differentiation, synergize with ROCK inhibition in enhancing cell proliferation and stem-cells-like features.

Different mechanisms have been shown to contribute to the CR of primary keratinocytes and airway epithelial cells, such as cell transition to S phase, enhancing cell cycle progression [136,144]. In their work, Liu and colleagues reported that the overall keratinocyte CR process relies on the combination of F medium, feeders, and the ROCK inhibitor. It is important to observe that ROCK inhibitor significantly extends keratinocyte life span but does not lead to indefinite proliferation in absence of feeder cells. In this context, it has been shown that irradiation of the J2 feeder cells is necessary for CR since the release of diffusible factors by dying feeder cells provides essential signals that act in parallel with Y-27632 [145]. Interestingly, Ligaba and colleagues performed a screening and showed that J2 FL secreted factors that stimulate cell growth by modulating TGF-β signaling, SMADs pathway, F-actin organization, and cytoskeleton modifications [144].

Furthermore, Mondal and colleagues suggested that the feeder component of the CR cocktail can induce in keratinocytes an elevated expression of a natural p53 isoform (∆133p53α), which enhances hTERT expression and correlates with an elevated telomerase activity and cell-proliferation potential [146], highlighting the essential contribution of FL to CR. However, Y-27632 addition is required for indefinite expansion of cells even upon overexpression of ectopic ∆133p53α.

Dakic and colleagues showed instead that human foreskin keratinocytes can be indefinitely propagated in the absence of FL without changes in the expression level of hTERT upon cMyc overexpression and a concomitant Y-27632 treatment [147]. In this context, the main effect of Y-27632 appears to be related to the suppression of the apoptotic program, which is typically engaged following cMyc overexpression.

Therefore, a growing evidence underlines that CR method applied to patient-derived cell expansion can be a powerful tool in regenerative medicine and tissue engineering. 

Multiple points of cross-talk between the mTORC1/mTORC2 and the ROCK pathways have been identified in different cellular contexts, including epidermal keratinocytes, in which mTORC1 disruption leads to hyperactivation of ROCK [95]. Considering the aforementioned effects of a single inhibition of mTOR or ROCK pathway on keratinocyte expansion, an outstanding question is whether dual mTOR/ROCK inhibition may favor long-term clonal expansion and stem cells maintenance in keratinocytes. An indication that this strategy may be beneficial in the context of keratinocyte expansion ex vivo can be found in work by Horvath and colleagues. They found that rapamycin treatment can “rejuvenate” the human keratinocyte DNA methylation profile independently of replicative senescence, whereas Y-27632 did not. However, rapamycin indeed delayed the onset of keratinocyte senescence and differentiation, and the combination of rapamycin and Y-27632 showed that cells maintained a “younger” epigenetic profile despite an acceleration of their cell cycle. Thus, the proliferation-promoting property of Y-27632 had a stronger impact on cell-proliferation rates as compared to the mild cytostatic effect of rapamycin, leading to an overall gain in cell proliferation [148]. However, the effects of the combinatorial use of mTOR and ROCK inhibitors were evaluated only on primary human newborn keratinocyte cultures derived from three newborn individuals, and the effects of the combinatorial treatment on adult keratinocytes were not assessed. 

Table 1 summarizes the main effects of the use of mTOR and ROCK inhibitors on cultured keratinocytes referenced in the text.

## 4. Conclusions and Outlook

In summary, both mTOR and ROCK inhibition favor the expansion of undifferentiated keratinocyte populations. The majority of reports suggest that in this context, mTOR- and ROCK-inhibitors operate via largely independent cellular mechanisms, with mTOR inhibition mostly proving effective in prevention of cell senescence, metabolic reprogramming, autophagy, stress protection, and epigenetic resetting, whereas ROCK inhibition acts primarily by preventing the execution of keratinocyte terminal differentiation program through effects on actin cytoskeleton dynamics and/or by forcing the balance between symmetric/versus asymmetric cell divisions. 

These two pharmacological approaches seem promising in the context of regenerative medicine applications of cultured keratinocytes, as they may enhance the expansion of undifferentiated cells, especially in the case of patients in which the endogenous stem cells pools are depauperated. However, approaches based on either mTOR or ROCK inhibition have potential drawbacks. For instance, rapamycin slows down keratinocyte proliferation, and this may significantly delay the production of confluent keratinocyte sheets for transplantation purposes in burn patients, in which a rapid expansion of cultured cells is highly desirable. In the context of CR based on ROCK inhibition, the resumption of proliferation in committed progenitor cells that would otherwise differentiate can accelerate the expansion of cultured keratinocytes, but it may also lead to a dilution of “authentic” holoclone forming cells within the culture. In this scenario, the rapid onset of terminal differentiation that follows the withdrawal of ROCK inhibitors upon transplantation may be especially detrimental for patients in which the number of holoclone forming cells is already compromised by aging or disease and who would mostly benefit from their expansion ex vivo.

Moreover, the long-term effects of both mTOR- and ROCK-inhibitor-based approaches upon xenotransplantation of human keratinocyte sheets in immune compromised animals have not been reported. The lack of these data currently precludes the transition towards a clinical use of keratinocytes expanded ex vivo in the presence of these drugs. 

**Figure 3:**
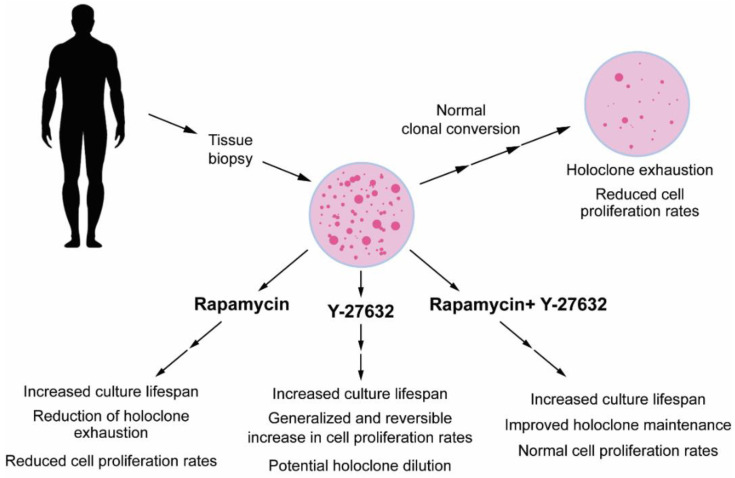
Summary of potential applications of rapamycin and Y-27632 in keratinocyte (stem cells) expansion for transplantation purposes. The diagram illustrates the normal process of clonal conversion and holoclone exhaustion occurring upon ex vivo expansion of keratinocytes isolated from patients’ stratified epithelia via a tissue biopsy. In the lower part of the figure are indicated advantages and disadvantages of the individual use of rapamycin and Y-27632 (see text and Table 1 for details) and the potential synergy of their combination for improving holoclone maintenance and expansion.

In light of the effectiveness of the 2i culture condition in maintaining authentic pluripotent ES cells, it would be important to test the combinatorial use of mTOR and ROCK inhibitors both in culture and after xenotransplantation. One attempt in this direction has been already made in human keratinocytes [148]. Although the long-term effects of this combination have not been fully explored, data suggest that the pro-proliferative effects of ROCK inhibition are not substantially affected by mTOR inhibition, and the “epigenetic rejuvenation” by rapamycin is still present with a concomitant ROCK pharmacological inhibition. Therefore, it is tempting to speculate that by suppressing different keratinocyte processes that lead to holoclone depletion, rapamycin, and Y-27632 may synergize in maintaining cultured keratinocyte stem cells functionality, with potential stem cell amplification above the therapeutic threshold that would not be otherwise obtained under standard culture conditions. Another potential strategy would be to use the inhibitors sequentially, starting from the use of rapamycin to amplify rare holoclone populations and then to proceed with Y-27623 for a rapid expansion of cells to avoid the overgrowth of committed keratinocyte populations that may dilute holoclone-forming cells in therapeutic grafts. The recent finding of the selective senolytic effect of rapamycin on cells with shortened telomeres suggests that its application to cultured keratinocytes may improve the selection of functional stem cells with the longest telomeres [85]. One further indication that argues in favor of a combinatorial use of rapamycin and Y-27623 is the evidence of an hyperactive ROCK signaling in keratinocytes in which mTORC1 signaling is suppressed [95]. Figure 3 summarizes potential advantages and disadvantages of the use of rapamycin and Y-27632 either alone or in combination.

It would be also important to determine whether a combinatorial use of mTOR and ROCK inhibitors can allow expansion of holoclones in the absence of a murine fibroblast feeder layer. Although the presence of feeder cells in keratinocyte cultures has never been associated per se with adverse events in clinical settings [5], the presence of a feeder layer of murine origin imposes a number of quality control procedures to comply with Good Manufacturing Practices rules, which raise the overall costs of production of keratinocyte autografts.

The elevated success rate of cultured keratinocyte transplantation procedures suggest that pharmacological manipulation of keratinocyte cultures may not be necessary in many cases (e.g., in cultures derived from young individuals). However, an improved holoclone maintenance and amplification would likely increase the number of patients suffering from skin and corneal diseases that might benefit from such procedures and for whom no other therapeutic option is available. This issue is of great actuality in light of the difficulties that have progressively emerged regarding the use of ES- or iPS cell derivatives in clinical trials, which are delaying the time in which these experimental procedures will become available to patients [150].

## Figures and Tables

**Table 1:** Summary of the effects of mTOR and ROCK inhibitors in cultured Keratinocytes.

Inhibitor	Cell Source	Feeder Layer	Effects on Cultured Cells	Article (First Author)
Rapamycin	Wnt1-overexpressing murine HFSCs	No	Reduced DNA damage, maintenance of stem cell markers	Castilho [87]
	Human oral keratinocytes	No	Increased clonogenicity and lifespan, protection from oxidative stress, reduced senescence, reduction of proliferation rate	Iglesias-Bartolome [90]
	Human corneal epithelial cells	No	Increased clonogenicity and stem cell markers expression, reduced senescence and apoptosis, reduced proliferation rate	Gidfar [93]
	Human skin keratinocytes	Yes	Reduced DNA methylation (epigenetic aging), reduced proliferation rate	Horvath [148]
Rapamycin, Torin, AZD8055	Normal murine skin keratinocytes	No	Reduced expression of desmosomal proteins and loss of cell–cell adhesion	Asrani [95]
Y-27632	Rheb-KO and Raptor-KO murine skin keratinocytes	No	Rescue of adhesion defects caused by mTORC1 deficiency	Asrani [95]
	Porcine airway epithelial cells	No	Unlimited increase of lifespan	Dale [149]
	Human skin keratinocytes	Yes	Unlimited increase of lifespan, increased cMyc expression, stabilization of telomeres length	Chapman [132]
	Human skin keratinocytes	Yes	Increased proliferation rate	Horvath [148]
	Human, bovine, and murine skin keratinocytes	Yes	Unlimited increase of lifespan, increased proliferation rate	Chapman [133]
	Immortalized and normal human skin keratinocytes	Yes	Inhibition of clonal conversion	Nanba [140]
	Human neonatal foreskin keratinocytes	No	Increased lifespan in low-calcium medium	Strudwick [134]
	Human neonatal foreskin keratinocytes	Yes	Increased cellularity of colonies with gain of cell proliferation	Roshan [141]
Fasudil, HA1000, GSK429286	Human skin keratinocytes	Yes	Unlimited increase of lifespan, increased proliferation rate	Chapman [133]
Rapamycin + Y-27632	Human skin keratinocytes	Yes	Reduced DNA methylation (epigenetic aging), normal proliferation rate	Horvath [148]
